# Structure of the PUB Domain from Ubiquitin Regulatory X Domain Protein 1 (UBXD1) and Its Interaction with the p97 AAA+ ATPase

**DOI:** 10.3390/biom9120876

**Published:** 2019-12-14

**Authors:** Mike Blueggel, Johannes van den Boom, Hemmo Meyer, Peter Bayer, Christine Beuck

**Affiliations:** 1 University of Duisburg-Essen, Structural and Medicinal Biochemistry, Centre for Medical Biotechnology (ZMB), 45117 Essen, Germany; mike.blueggel@uni-due.de (M.B.); peter.bayer@uni-due.de (P.B.); 2 University of Duisburg-Essen, Molecular Biology, 45117 Essen, Germany; Johannes.van-den-boom@uni-due.de (J.v.d.B.); Hemmo.meyer@uni-due.de (H.M.)

**Keywords:** UBXD1, p97/VCP/Cdc48, PUB domain, NMR structure, protein–protein interaction

## Abstract

AAA+ ATPase p97/valosin-containing protein (VCP)/Cdc48 is a key player in various cellular stress responses in which it unfolds ubiquitinated proteins to facilitate their degradation by the proteasome. P97 works in different cellular processes using alternative sets of cofactors and is implicated in multiple degenerative diseases. Ubiquitin regulatory X domain protein 1 (UBXD1) has been linked to pathogenesis and is unique amongst p97 cofactors because it interacts with both termini of p97. Its N-domain binds to the N-domain and N/D1 interface of p97 and regulates its ATPase activity. The PUB (peptide:*N*-glycanase and UBA or UBX-containing proteins) domain binds the p97 C-terminus, but how it controls p97 function is still unknown. Here we present the NMR structure of UBXD1-PUB together with binding studies, mutational analysis, and a model of UBXD1-PUB in complex with the p97 C-terminus. While the binding pocket is conserved among PUB domains, UBXD1-PUB features a unique loop and turn regions suggesting a role in coordinating interaction with downstream regulators and substrate processing

## 1. Introduction

The ubiquitin proteasome system (UPS) critically regulates various cellular stress response pathways including ER-associated degradation (ERAD), ribosomal quality control (RQC), DNA damage repair, and autophagy that ensure cellular and protein homeostasis. A key element of the UPS is the conserved AAA+ ATPase p97 (also called Cdc48 or VCP for valosin-containing protein) that targets a subset of ubiquitin-modified substrate proteins, extracts them from cellular structures, and unfolds them to facilitate degradation in the proteasome [1,2,3].

p97 comprises a regulatory N-terminal domain, two AAA ATPase domains (D1 and D2) that form a stacked hexamer, and a short, disordered C-terminal region [4,5]. Protein unfolding is mediated by ATP-driven translocation of substrate proteins through the central channel of the hexamer [6,7,8,9]. The function and activity of p97 is regulated by a large number of cofactor proteins [10]. They include substrate adapters that bind at the N-domain and assist insertion of the substrates into the D1 pore [9,11] and also many regulatory proteins that modulate p97 activity in different cellular processes [10].

Depletion of p97 is lethal, and point mutations in p97 cause a degenerative disease in humans that features inclusion body myopathy (IBM), fronto-temporal dementia (FTD), amyotrophic lateral sclerosis (ALS), and Paget’s disease (PD) of bone (collectively called IBMPFD/ALS) [12]. Most disease-associated mutations are located at the interface between the N and D1 domains [13]. They affect cofactor binding, including the interaction with the UBXD1 cofactor, suggesting a link to pathogenesis [14,15]. UBXD1 is involved in p97-mediated functions in autophagy of damaged lysosomes and mitochondria as well as in WNT signaling [16,17,18]. Of note, UBXD1 is unique amongst the p97 cofactors because it is able to interact with both termini of p97 [19]. The N-terminus of UBXD1 includes a VCP-interacting motif (VIM) and additional elements that bind to the N/D1 interface of p97 [20,21]. This interaction regulates p97 ATPase activity by modulating the up/down mobility of the p97-N domain, and this regulation is affected by disease mutations [21,22].

Like many p97 cofactors, UBXD1 also possesses a C-terminal ubiquitin regulatory X (UBX) domain, which, however, lacks a critical FP motif and does not contribute to p97 binding [23]. Instead, the second element of the two-pronged interaction mechanism is a PUB (peptide:*N*-glycanase and UBA or UBX-containing proteins) domain [24], which binds the C-terminal tail of p97, possibly close to the D2 pore where unfolded proteins exit the central channel of p97. So far, the implications of this domain in substrate processing and its implication in disease pathogenesis are unknown.

PUB domains have been found in several proteins associated with the UPS that often also contain ubiquitin-associated (UBA) or UBX domains. The PUB domains were first considered as p97 binding modules [23,25,26], but since the HOIL-1-interacting protein (HOIP/RNF31) also uses its PUB domain to interact with the OTU deubiquitinase with linear linkage specificity (OTULIN) and spermatogenesis-associated protein 2 (SPATA2) [27,28], PUB domains adopt a broader function within the UPS. Peptide *N*-glycanase (PNGase) binds to the PUB interacting motif (PIM) in the p97 C-terminus via its PUB domain and removes N-linked oligosaccharide chains from misfolded glycoproteins prior to their degradation by the proteasome [29,30,31]. The E3 ubiquitin ligase HOIP/RNF31 associates with the proteins HOIL-1 and SHARPIN to form the linear ubiquitin assembly complex (LUBAC) which attaches linear M1-linked ubiquitin chains to its substrates and regulates inflammation via the NF-κB pathway [32,33,34]. The LUBAC is regulated by the linear, chain-specific deubiquitinases OTULIN and CYLD. OTULIN contains a PIM sequence and is bound directly by HOIP-PUB [35,36]. CYLD, however, does not interact directly with HOIP, but via the adaptor protein SPATA2 [28,37]. SPATA2 contains both a PIM sequence, which is tightly bound by HOIP-PUB, and also a PUB domain on its own, which cannot bind a canonical PIM sequence but was found to interact with the ubiquitin-specific protease (USP) domain of CYLD [28,37]. Furthermore, HOIP also binds the p97 PIM, albeit with lesser affinity, and has been shown via co-immunoprecipitation/mass spectrometry experiments to interact with p97 in cells [34]. While p97 has also been associated with the NF-κB pathway [38,39], it is still not clear whether it is recruited via its interaction with HOIP [34].

While a few structures of other PUB domains are known [25,27,28,29,34] and conserved residues involved in PIM binding have been identified, the interesting question lies in the differences between homologous domains, which will lead the way to understand their functional role in the downstream processing of p97 substrates.

Here we present the solution structure of the UBXD1-PUB domain together with binding studies, mutational analysis, and a model of the UBXD1-PUB/p97-C complex. The structure of the UBXD1-PUB domain sheds light onto differences to other PUB domains and serves as an important building block to further understand the unique role of UBXD1 and its interaction with p97.

## 2. Materials and Methods

### 2.1. Cloning, Protein Expression, and Purification

N-terminal hexahistidine-tagged UBXD1-PUB (150–264) was cloned into a pET28a expression vector using the NdeI and HindIII restriction sites. UBXD1 point mutants were produced by site-directed mutagenesis following the Quikchange protocol (Agilent Technologies). All primer sequences are listed in Table S1.

In short, all proteins were expressed in *Escherichia coli* Rosetta2 in M9 minimal media with 50 µg/mL kanamycin and 30 µg/mL chloramphenicol and purified on a nickel-NTA affinity column followed by size exclusion chromatography after thrombin cleavage of the tag.

For the expression of unlabeled protein, 50 mL of an overnight culture in M9 minimal media was harvested, resuspended in 1 L M9 medium, and grown up to OD_600_ = 1.0 at 37 °C, 160 rpm. For the expressions of ^15^N or ^13^C,^15^N isotopically labeled protein, 1 l M9 minimal medium supplemented with 0.6 g/L (^15^N) ammonium sulfate and, for ^13^C,^15^N labeling, 3 g/L (^13^C) glucose were used and grown up to OD_600_ = 1.0. After induction of protein expression with 0.1 mM Isopropyl-β-D-thiogalactopyranosid (IPTG), cells were incubated for 4 h at 37 °C followed by centrifugation (4000 rpm, 20 min, 4 °C). Pelleted cells were dissolved in PBS buffer, pH 7.4, supplemented with 1 mM PMSF, and lysed with lysozyme (1 mg/mL) in combination with sonication. Upon ultracentrifugation (35,000 rpm, 60 min, 4 °C) to clear the lysate, the protein was purified by Ni-NTA affinity chromatography with PBS buffer, pH 7.4, and eluted with an imidazole gradient. The His_6_ tag was cleaved with thrombin protease prior to size exclusion chromatography in 50 mM sodium potassium phosphate, 150 mM sodium chloride, pH 6.5. The protein was concentrated, and the buffer exchanged to sodium potassium phosphate buffer (50 mM NaKP_i_, pH 6.5), using a centrifugal concentrator (Vivaspin) with a 5 kDa molecular weight cutoff. To the NMR samples in H_2_O, 10% D_2_O was added. To prepare NMR samples in 100% D_2_O, the protein in buffer was lyophilized and resuspended in 100% D_2_O. The molecular weight of the protein was confirmed by MALDI-MS.

### 2.2. Peptides

Unlabeled p97-C10 (TEDNDDDLYG), p97-C13 (SVYTEDNDDDLYG), and phosphorylated p97-C13 (SVYTEDNDDDLpYG) peptides for NMR and ITC as well as 5,6-FAM-labeled peptides for fluorescence studies were purchased from Caslo ApS (Denmark).

### 2.3. Circular Dichroism (CD) Spectroscopy

CD spectra were recorded on a Jasco J-170 CD spectrometer in a 1 mm quartz cuvette at 25 °C averaging 25 scans. Samples consisted of 0.15 mg/mL protein in 50 mM NaKPi, pH 6.5. The secondary structure composition was analyzed using DichroWeb (http://dichroweb.cryst.bbk.ac.uk) employing the CDSSTR algorithm [40,41].

### 2.4. MALDI-TOF Mass Spectrometry

Samples for MALDI-TOF-MS were desalted using C18-pipette tips (Supelco) according to the manufacturer’s protocol and mixed with a matrix containing 2,5-dihydroxyacetone (2,5-DHAP) and diammoniumhydrogencitrate (DAC). MALDI-MS spectra were measured with a Bruker Autoflex speed mass spectrometer, resulting in the measured mass of 13.84 kDa (M+K^+^; theoretical MW = 13.80 kDa).

### 2.5. Nuclear Magnetic Resonance (NMR) Spectroscopy

The NMR samples contained 200–800 µM of ^15^N,^13^C- or ^15^N-labeled protein in 50 mM NaKP_i_ pH 6.5, 90%/10% (v/v) H_2_O/D_2_O. ^13^C(aliphatic)-COSY and TOCSY experiments were measured in the same buffer with 100% D_2_O. All spectra were recorded at 25 °C on a 700 MHz Bruker Ultrashield NMR spectrometer equipped with a 5 mm TCI (^1^H/^13^C/^15^N) triple resonance cryoprobe head. Spectra were processed with Topspin 3.5 (Bruker) and analyzed with CARA [42].

#### 2.5.1. NMR Assignments

Backbone assignments of ^15^N,^13^C-PUB were accomplished using classical triple-resonance experiments [43]. Side chain assignments were based on 3D H(CCCO)NH, CC(CO)NH, as well as 3D aliphatic HC(C)H-COSY and ^13^C(aliphatic)- and ^13^C(aromatic)-resolved HC(C)H-TOCSY (DIPSI3 mixing time 10.9 ms) experiments. To assign the aromatic resonances a ^13^C(aliphatic),^13^C(aromatic)-resolved HC(C)H-TOCSY [44] was recorded with a FLOPSY2 mixing time of 28.87 ms. Interproton distance constraints were obtained from 3D ^15^N-, ^13^C(aliphatic)-, and ^13^C(aromatic)-resolved NOESY spectra recorded with a mixing time of 100 ms.

Protein chemical shifts were referenced to internal 2,2-dimethyl-2-silapentane-5-sulfonate sodium salt (DSS). The ^15^N and ^13^C chemical shifts were referenced indirectly to DSS according to IUPAC recommendations [45].

#### 2.5.2. NMR Structure Calculation

Torsion angle restrains were calculated with TALOS [46]. Hydrogen bond donors could not be derived from H/D-exchange NMR experiments because the proton exchange was too fast. Therefore, H-bonds within secondary structure elements were derived from the chemical shift index (CSI) [47], dihedral angles obtained from TALOS, and a backbone structural model calculated from C_α_ and C_β_ chemical shifts by CS-Rosetta [48]. The use of H-bond restraints improved the quality of the resulting structure. A structure calculation without H-bond restraints was performed to ensure that no artificial bias was introduced.

NOESY peak picking, assignment, and the structure calculation was performed with the ATNOS/CANDID module of the UNIO 10 software [49,50,51] in conjunction with CYANA 3.98 [52]. The 20 conformers with the lowest residual CYANA target function values obtained from the seventh ATNOS/CANDID/CYANA cycle were subjected to energy minimization in a water shell with YASARA. After structure validation, 20 conformers were selected to represent the NMR structures, and the programs PyMol and UCSF Chimera [53] were used to analyze this ensemble of conformers.

The accession number for the apo structure of UBXD1-PUB reported in this paper is PDB # 6SAP, and the NMR chemical shifts are available in the BMRB database under the accession number 27977. The coordinates of the model shown in Figure 4 are available on request.

Structure validation. The PSVS server (http://psus-1_5-dev.nesg.org) and the PDB validation server (https://validate-pdbe.wwpdb.org/validservice/) were used to analyze the stereochemical quality of the molecular structures.

#### 2.5.3. NMR Titration Experiments

To 362 μM ^15^N-labeled UBXD1-PUB, the p97-C10 or p97-C13 peptides were added stepwise to a final concentration of 1.1 mM. For the titration of the phosphorylated p97-C13-pY^805^ peptide to the PUB domain, the protein concentration was 270 µM, and the peptide was titrated to a final concentration of 1.2 mM. After each addition, the ^1^H,^15^N-HSQC spectrum was recorded.

The chemical shift perturbation Δδ_total_ was calculated from the ^1^H- and ^15^N-shifts according to Equation (1) [54] using the spectra with no peptide and the highest concentration of peptide, where Δδ_N_ and Δδ_H_ represent the chemical shift perturbation value of the amide nitrogen and proton, respectively:

(1) $$\Delta \delta_{\mathrm{total}}=\sqrt{{\left(\Delta \delta_{H}\right)}^{2}+{\left(0.154\cdot \Delta \delta_{N}\right)}^{2}}.$$

#### 2.5.4. ^15^N Relaxation Measurements

Nitrogen T_1_, T_2_ and steady state heteronuclear NOE relaxation experiments for ^15^N-UBXD1-PUB were collected at 25 °C using sensitivity-enhanced pulse programs [55]. T_1_ and T_2_ relaxation delay times ranged from 20 to 1400 and 10 to 190 ms, respectively. The saturated and unsaturated heteronuclear NOE experiments were collected in an interleaved manner, and the ratio of signal intensities was calculated for each resonance. T_1_ and T_2_ were fitted to a monoexponential function [56] using GraphPad Prism 5.0. The rotational correlation time τ_c_ for each residue was calculated from T_1_/T_2_ (with ν_N_ = Larmor frequency of nitrogen) [57]:

(2) $$\tau_{c}=\frac{1}{4\cdot \pi \cdot \nu_{N}}\cdot \sqrt{6\cdot \frac{T_{1}}{T_{2}}-7}.$$

The molecular weight (MW) in solution of 13.0 kDa was obtained from the linear correlation between MW and τ_c_ as determined by Rossi et al. [57] and the averaged τ_c_ = 7.9 ns.

### 2.6. Fluorescence Anisotropy

Fluorescence anisotropy measurements (excitation: 495 nm; emission 518 nm) were carried out on a JASCO FP-8300 fluorescence spectrometer in 50 mM NaKP_i_, pH 6.5 at 25 °C, titrating PUB constructs to 5,6-FAM labeled p97-C10 peptide (100 nM). All titrations were performed in triplicate. Binding curves of the averaged data were fitted with GraphPad Prism 5.0 (GraphPad) using the quadratic binding equation for a one-site specific binding model:

(3) $$r=r_{0}+r_{\max}\cdot \frac{\left(F+x+K_{D}\right)-\sqrt{{\left(F+x+K_{D}\right)}^{2}-4\cdot x\cdot F}}{2\cdot F},$$

with r = anisotropy, r_0_ = anisotropy without protein, r_max_ = maximum anisotropy, F = fluorescent probe (labeled peptide or protein) concentration, x = titrant protein concentration, and K_D_ = dissociation constant.

Data points are shown as means ± standard deviation based on the three experiments. K_D_ values are given as fit of the averaged data points ± standard deviation of the fit.

### 2.7. Isothermal Titration Calorimetry (ITC)

ITC measurements were performed in triplicate with a MicroCal iTC2000 (Malvern Pananalytical). UBXD1-PUB (100 µM) was titrated with 1 mM of p97-C10 peptide in 50 mM NaKPi buffer (pH 6.5) at 25 °C in 40 steps with a volume of 0.5 µL for the first 9 steps and 1.0 µL for the remaining ones. The binding parameters were calculated using MicroCal Origin (OriginLab) by fitting the data to a single-site binding model, and the binding parameters were averaged, yielding K_D_ = 15 ± 5 µM, a stoichiometry of n = 1.1 ± 0.2, and the thermodynamic parameters ΔH = −3255 ± 483 cal/mol and ΔS = 10 cal/mol/K.

### 2.8. Alignment of PUB structures and RMSD Calculation

The apo structures of the UBXD1, PNGase, HOIP, and SPATA2 PUB domains were aligned in PyMol with the cealign command, using the core PUB residues 153–261 for UBXD1, 14–107 for PNGase (pdb # 2CCQ, [29]), 52–155 for HOIP (pdb # 4OYJ, [27]), and 64–168 for SPATA2 (pdb # 5LJM, [28]).

### 2.9. Model of UBXD1-PUB/p97-PIM Complex

The UBXD1-PUB model from the NMR ensemble with the most open PIM binding pocket (model #14) was aligned with the PNGase-PUB structure in complex with the p97-C10 peptide (pdb # 2HPL) in PyMol. In this orientation, the p97-PIM peptide from the PNGase complex structure was added to the UBXD1-PUB apo structure. The peptide comprises only the last 5 residues (D802–G806) because the preceding residues were disordered and, thus, not visible in the crystal structure [25]. The resulting UBXD1-PUB7p97-PIM model was then subjected to energy minimization in Yasara using the macro em_run.

## 3. Results

### 3.1. NMR Solution Structure of UBXD1-PUB.

**Figure 1:**
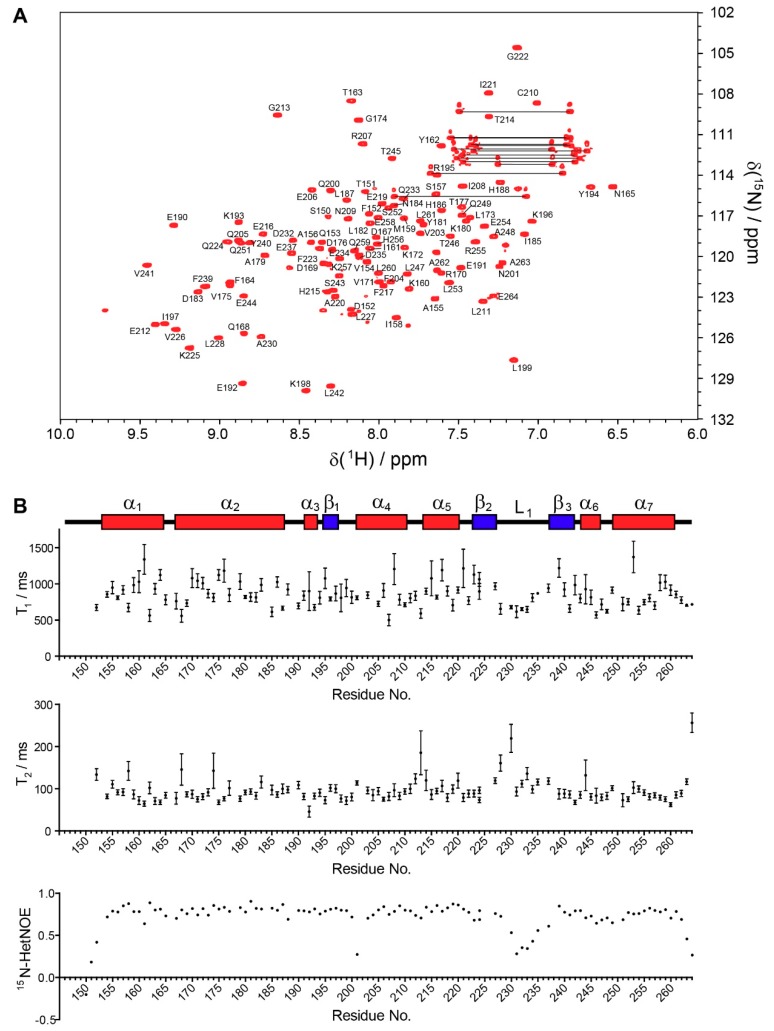
^15^N-HSQC spectrum and ^15^N relaxation data of UBXD1-PUB. (**A**) ^15^N-HSQC spectrum with amide assignments; (**B**) ^15^N relaxation data (top: T_1_ relaxation times, middle: T_2_ relaxation times, bottom: ^15^N-HetNOE) plotted against the UBXD1-PUB sequence. Secondary structure elements are shown above.

The UBXD1-PUB domain was previously identified as functional p97 binding unit [19,29], and the construct ranging from residues 150–264 yielded a well-dispersed ^15^N-HSQC spectrum (Figure 1A). The averaged rotational correlation time of τ_c_ = 7.9 ns is obtained from the ratio of the ^15^N longitudinal (T_1_) and transverse (T_2_) relaxation times of all amide resonances (Figure 1B). τ_c_ can be correlated to the molecular weight (MW) of the protein in solution and reveals whether a protein forms multimeric assemblies [57]. For UBXD1-PUB, this yielded a MW of 13.0 kDa, which corresponds to the size of a monomer. The ^15^N-HetNOE experiment, which shows whether an amide is situated in a structured or flexible region of the protein, revealed that the whole domain, except one loop region between P229 and P236, was a rigid unit with little internal mobility (Figure 1B).

**Figure 2:**
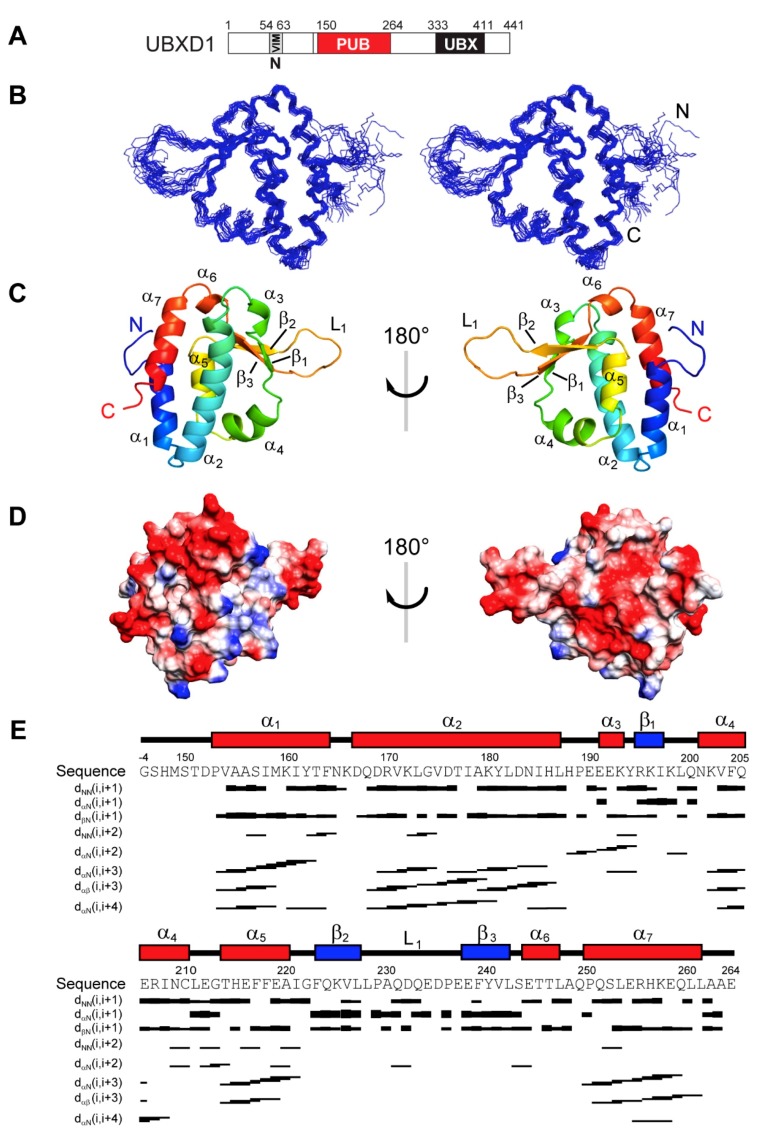
NMR structure of the UBXD1-PUB domain. (**A**) Schematic representation of UBXD1 with indication of the known functional domains. The UBXD1-PUB construct used in this study comprises residues 150–264; (**B**) bundle of 20 NMR conformers showing backbone atoms only in stereo view; (**C**) cartoon representation of the UBXD1-PUB domain, front and back view; (**D**) electrostatic charge distribution on the UBXD1-PUB domain, front and back view. Positively charged areas are shown in blue and negatively charged in red (−8 to +8 kcal/(mol·e)); (**E**) NOE distribution chart. The UBXD1-PUB sequence and secondary structure elements are shown above.

NMR assignments were obtained from 3D triple resonance and HCCH-COSY and -TOCSY spectra. ^13^C and ^15^N NOESY spectra yielded 1221 distance constraints that, together with 567 dihedral angle constraints, were used to generate a bundle of the 20 lowest-energy conformers representing the NMR structure of UBXD1-PUB (Figure 2; for statistics see Table 1).

UBXD1-PUB consists of an antiparallel four-helix bundle comprising helices α_1_ (P153-F164), α_2_ (Q168-L187), α_5_ (T214-A220), and α_7_ (P250-L261). The β-strands β_1_ (K196-L198), β_2_ (F223-L227), and β_3_ (E238-L242) form an antiparallel β-sheet packed against the helical bundle with an extended loop L1 (P229-P236) between strands β_2_ and β_3_. The short 3_10_-helix α_3_ (E192-E194) and α_4_ (K202-C210) together with sheet β_1_ are part of the extended connection looping around between helices α_2_ and α_5_. The short helix α_6_ (E244-L247) connects sheet β_3_ and the C-terminal helix α_7_. The overall structure shares high similarity with the core PUB fold of PNGase [25,29], which is also found in the PUB domains of HOIP [27,34] and SPATA2 [28]. HOIP and SPATA2, however, feature extended PUB domains with additional N- and/or C-terminal helices. In contrast to the other PUB domains, UBXD1-PUB displays the unique extended loop L1, which might be responsible for specific protein–protein interactions.

### 3.2. Interaction of UBXD1-PUB with p97-PIM.

**Figure 3:**
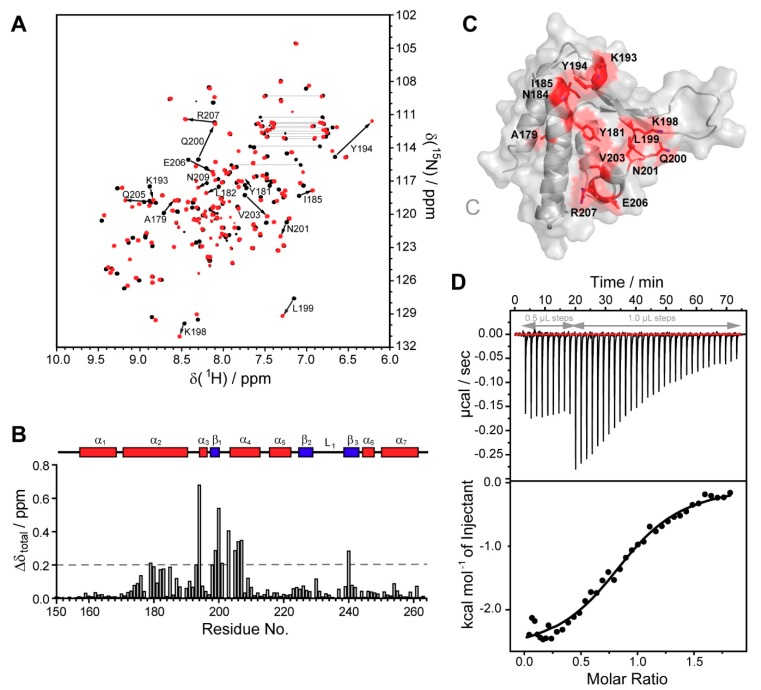
UBXD1-PUB binding to the p97 PIM motif. (**A**) ^15^N-HSQC titration of ^15^N-labeled UBXD1-PUB with p97-C10 PIM peptide. The spectrum in the absence of PIM is shown in black and the one in the presence of 1.1 mM p97-PIM in red. Peaks shift during the titration, indicating intermediate-to-fast-exchange (see supplemental Figure S1 for full titration data). The residues experiencing the largest perturbations are labeled, and the signal shifts are indicated with an arrow; (**B**) chemical shift perturbations Δδ_total_ plotted against the UBXD1-PUB sequence. The dotted line represents the Δδ_total_ average. The secondary structure elements are shown above; (**C**) mapping of residues with larger-than-average shifts (>0.2 ppm, highlighted in red) onto the UBXD1-PUB structure; (**D**) representative ITC experiment of p97-C10 peptide titrated to UBXD1-PUB wt. The initial titration steps were executed with a volume of 0.5 µL, all subsequent steps with 1 µL.

It was demonstrated previously that the UBXD1-PUB domain interacts with the unstructured C-terminal residues of p97 [19,27,29]. In our hands, isothermal titration calorimetry (ITC) experiments showed that the peptide comprising the last 10 residues of p97 (p97–C10) interacts with UBXD1-PUB with a 1:1 stoichiometry (n = 1.1 ± 0.2) and a K_D_ of 15 ± 5 µM, ΔH = -3255 ± 483 cal/mol, and ΔS = 10 cal/mol/K. The dissociation constant was confirmed by fluorescence anisotropy titrations (K_D_ = 14 ± 1 µM) and agreed with published values (Figure 3 and Figure 5C). This affinity was consistent with the K_D_ value of 12 µM reported by Elliot et al. [27] and was in the same low µM range as the affinities of homologous PUB domains for the p97-PIM peptide [27,28].

^15^N-HSQC NMR titrations are uniquely suited to determine the residues involved in the interaction. Titration of ^15^N-UBXD1-PUB with the p97-C10 and p97-C13 peptide showed large (>0.2 ppm) chemical shift perturbations (CSPs) of resonances assigned to amide groups residing in the region between the second and fourth α-helix (Figure 3 and Figure S1). The interacting residues V175, A179, K180, Y181, L182, I185, L187, E191, K193, Y194, K198, L199, Q200, N201, V203, E206, R207, N209, A230, F239, E258, and L261 are mapped onto the PUB structure in Figure 3. Residues Y181, L182, I185, Y 194, and I197 form a hydrophobic pocket, which is conserved in the PUB family. In the crystal structures of PNGase [25] and HOIP [34] in complex with the p97-PIM peptide, this pocket accommodates the conserved Tyr residue within the PIM motif.

In contrast to the unmodified p97-PIM, the p97-C13 peptide carrying a phosphorylated pY805 did not show any binding in a ^15^N-HSQC NMR titration up to a concentration of 1.25 mM (Figure S1B), which is consistent with phosphorylation of p97-Y805 also completely abolishing binding of the PNGase and HOIP PUB domains [25,34].

### 3.3. Structural Model of the UBXD1-PUB/p97-PIM Complex.

**Figure 4:**
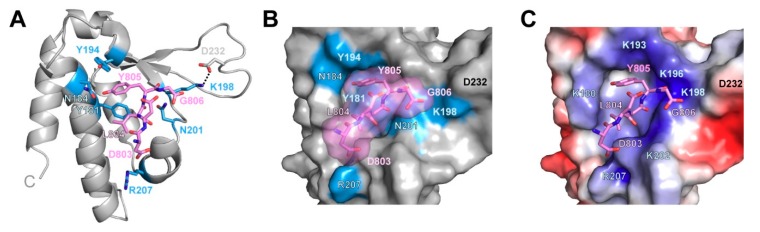
Model of the UBXD1-PUB/p97-PIM complex. (**A**) Energy-minimized model of the UBXD1-PUB/p97-PIM complex. UBXD1-PUB is shown as cartoon with the residues important for PIM binding highlighted as light blue sticks and the p97-PIM (residues 803–806) as pink sticks; (**B**) surface representation of the UBXD1 PIM binding pocket with bound p97-PIM. (**C**) Electrostatic charge distribution of the same view as in (**B**). Positively charged areas are shown in blue and negatively charged in red (−4 to +4 kcal/(mol·e)). Positively charged PUB residues are labeled in light blue.

Since the PIM binding pocket is highly conserved amongst PUB domains [28,34], and the crystal structure of PNGase-PUB in complex with the p97-PIM is known, we calculated a model of the UBXD1-PUB/p97-PIM complex by aligning the proteins, adding the PIM peptide to the UBXD1-PUB apo structure and performing an energy minimization in silico. This resulted in a slight adjustment of a few side chains compared to the apo structure in order to fully open the binding pocket (Figure 4). Within the hydrophobic pocket, p97-Y805 is stabilized by face-to-edge stacking in between Y181 and Y194 as well as putative H bonds of its OH group to the backbone carbonyl of Y181 and the side chain of N184. The carboxyl terminus of p97-G806 is situated within H bonding distance to the side chain of N201. K198 does not contact the carboxyl group of p97-G806 in our model, but it maintains an intramolecular salt bridge to D232 within the L1 loop as observed in the apo structure. p97-L804 is cradled by Y181, V203, and the aliphatic portion of the K180 side chain. The overall positively charged rim of the binding pocket, including R207 as well as K180, K193, K196, K198, and K202 (Figure 4C), ensures not only binding of the C-terminal carboxylate but also electrostatic attraction to the numerous acidic residues (^796^TEDNDDDLYG^806^) within p97-C10.

Overall, the PUB/p97-PIM complex is stabilized by a combination of hydrophobic and electrostatic interactions, with key interactions between the side chains of Y181, N184, and Y194 and the aromatic side chain of p97-Y805 inside a hydrophobic pocket of the PUB domain and binding of the free p97 C-terminus via H bonds and electrostatic interactions.

### 3.4. Structure-Based Mutational Analysis Confirms Conserved Binding Pocket.

**Figure 5:**
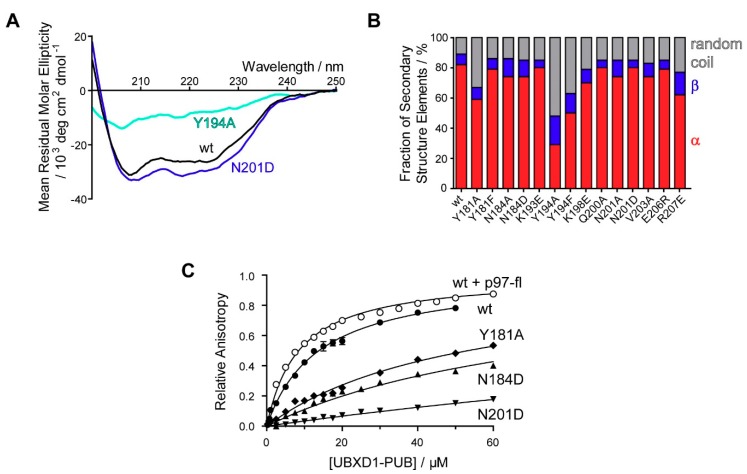
Mutational analysis of the UBXD1-PUB PIM binding pocket. (**A**) CD spectra of UBXD1-PUB wt and selected mutants; (**B**) secondary structure composition of UBXD1-PUB mutants obtained from deconvolution of the CD spectra with DichroWeb. The percentage of α-helices is shown in red, β-sheets in blue, and random coil in gray. While most mutants remain folded with a secondary structure composition similar to the wild type, the Y194A mutant shows a partial loss of secondary structure. (**C**) Fluorescence anisotropy binding curves for selected UBXD1-PUB constructs with 5,6-FAM labeled p97-C10 peptide or full length p97. Error bars represent the standard deviation of three experiments. Only the concentration range up to 60 µM is shown (for full titration data see Figure S2).

To evaluate the contribution of each residue to p97 binding, nine residues were selected for mutational analysis based on the NMR titration and the model of the UBXD1-PUB/p97-PIM complex. Correct folding of the mutants was confirmed using CD spectroscopy (Figure 5). The binding constants for each point mutant with p97-C10 were measured by fluorescence anisotropy (Table 2 and Figure S2). The largest decrease in binding affinity compared to the wild type PUB domain (K_D_ = 14 ± 1 µM) was observed for the mutants N201D, Y194F, and Y181A.

According to the model of the UBXD1-PUB/p97-PIM complex (Section 3.3 and Figure 4), N201 is involved in contacting the negatively charged C-terminal carboxylate of p97-G806. Thus, changing the neutral N to a negatively charged D results in electrostatic repulsion and a 20-fold increase in K_D_, while the corresponding N201A mutant, which lacks the ability to form a H-bond, shows a 5-fold higher K_D_.

Both Tyr residues Y194 and Y181 are conserved and essential for the recognition of p97-Y805 [30]. Mutating Y181 to A (K_D_ = 111 ± 2 µM) results in a much larger effect on p97 binding compared to the F mutant (K_D_ = 28.4 ± 0.6 µM), indicating that aromatic ring stacking is in fact crucial for the interaction, as predicted from our complex model (Figure 4).

Both Y194F and Y194A mutations also result in a large decrease in affinity. However, since both mutations, especially Y194A, cause a decrease in α-helical content revealed by their CD spectra (wt: 80%, Y194A: 30%, Y194F: 50%), the absolute affinities have to be viewed with a grain of salt. The OH group of Y194 is situated in H bonding distance (2.9 Å) to E191, which in turn is stabilized by an additional putative H bond to H188, in the loop region between helices α_2_ and α_3_, which might explain a destabilizing effect on the tertiary structure when it is removed (Figure 7 and Figure S3).

Mutating R207 to E results in a 6-fold decreased affinity, likely because this residue contacts one of the numerous aspartate residues within p97-C10. In contrast, K193E and Q200A do not show a decreased affinity compared to the wild type. Both their side chains face away from the binding pocket into the solvent; however, their amide groups are closer to the bound p97-C10, which explains the signal shifts observed in the NMR titrations.

## 4. Discussion

### 4.1. Structural Homology between PUB Domains and Unique Features of UBXD1-PUB

The UBXD1-PUB structure presented here (Figure 2) most closely resembles the PNGase PUB domain [29] (C_α_-RMSD = 2.01 Å), which defines the canonical PUB fold consisting of an antiparallel four-helix bundle, a short three-stranded β-sheet, as well as a 3_10_-helix α_3_ and another short helix α_4_ framing the β_1_ strand. The core PUB regions of HOIP (residues 52–155) and SPATA2 (residues 64–168) show a larger C_α_-RMSD of 3.42 or 4.09 Å, respectively.

**Figure 6:**
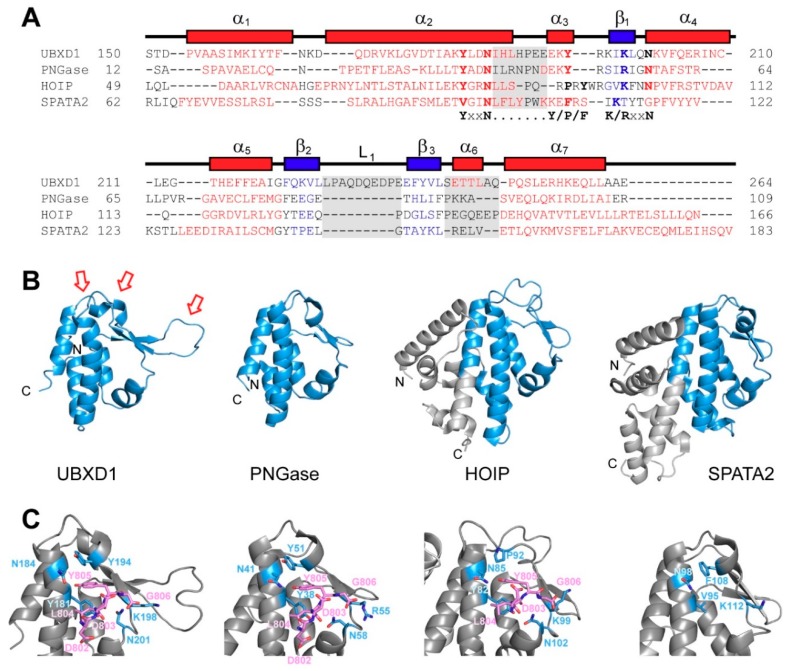
Comparison of UBXD1-PUB with other PUB domains. (**A**) Sequence alignment of UBXD1-PUB with the core PUB domains of human PNGase, HOIP, and SPATA2. The secondary structure of UBXD1-PUB is shown above (helices in red, β-sheets in blue). The conserved residues of the PIM pocket are in bold, and their motifs are labeled below. The loop and turn regions with the largest variability are highlighted with gray squares; (**B**) structural comparison of UBXD1-PUB with the PUB domains of PNGase (pdb # 2CCQ, [29]), HOIP (pdb # 4OYJ, [27]), and SPATA2 (pdb # 5LJM, [28]). The core PUB fold is shown in blue and the extensions of HOIP and SPATA2 in gray. Red arrows highlight the places where UBXD1-PUB differs most from the PUB consensus (gray boxes in A); (**C**) close-up of the PIM pockets (light blue, sticks) of UBXD1, PNGase (pdb # 2HPL, [25]) and HOIP (pdb # 4P0A, [34]) in complex with the p97-PIM peptide (pink), and SPATA2 (apo, pdb # 5LJM, [28]).

UBXD1-PUB features a unique extended L1 loop between the strands β_2_ and β_3_, where the PUB domains of PNGase, HOIP, and SPATA2 only present a short turn (Figure 6). Further differences between PUB domains are located at the top of the four-helix bundle, where UBXD1-PUB exhibits an additional short helix α_6_. These unique structural features of UBXD1-PUB might indicate additional protein interaction sites with other components of p97- and UBXD1-mediated reactions (further discussion see below).

### 4.2. Determinants of PIM Binding

The hydrophobic PIM binding pocket of UBXD1-PUB (Figure 2 and Figure 3) closely resembles the conserved Φ-Ψ pockets of PNGase and HOIP [25,27,28,29,34], which also bind canonical PIM sequences. The two conserved tyrosine residues (Y181 and Y194 in UBXD1) stack with the central PIM tyrosine (p97-Y805) in a face-to-edge manner. Furthermore, a conserved asparagine residue (UBXD1-N184) is in hydrogen bonding distance to the hydroxyl group of the PIM tyrosine. Taken together, this essential YxxN(x)_9_Y motif shared by UBXD1 and PNGase forms the core of the pocket, and mutation of any of these residues results in strongly impaired PIM binding.

**Figure 7:**
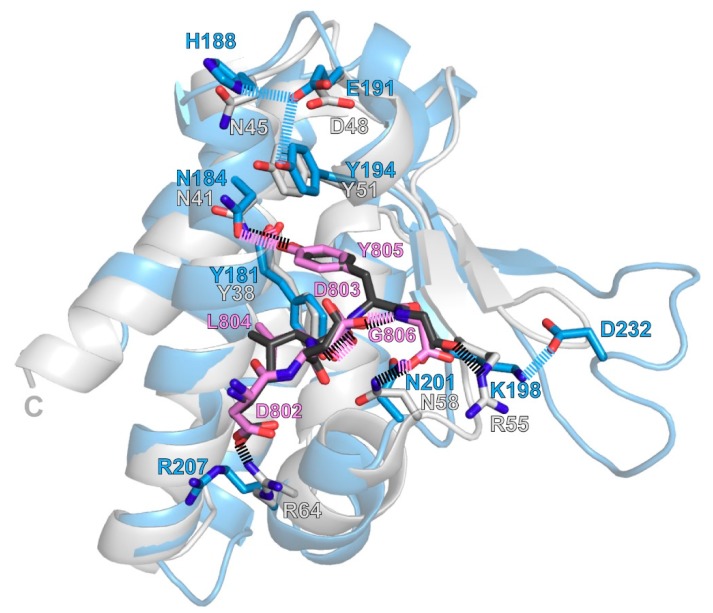
Comparison of the UBXD1-PUB/p97-PIM model with the PNGase/p97-PIM crystal structure (pdb # 2HPL [25]). The model of UBXD1-PUB is shown in blue with the PIM peptide model in pink. PNGase-PUB is shown in gray with the bound p97-PIM peptide in black. Important residues are shown as sticks and labeled in the respective color of each protein. Residues of the p97-PIM peptide are labeled in pink. Potential intermolecular H bonds and salt bridges are shown as dashed lines in the color of the respective peptide. Intramolecular H bonds and salt bridges that are present in UBXD1-PUB but not PNGase-PUB are shown as blue dashed lines.

The PIM binding pockets of UBXD1-PUB and its closest homologue PNGase-PUB and the position of the p97-PIM peptide inside the pocket are almost identical, with small differences (Figure 6 and Figure 7). The side chains of the conserved UBXD1-Y181 and PNGase-Y38 are in the same position, stacking with p97-Y805 and forming a putative H-bond (3.2 or 3.1 Å, respectively) to the backbone carbonyl of p97-L804. UBDXD1-Y194 and PNGase-Y51, which represent the second conserved tyrosine of the PIM pocket, both stack with p97-Y805 and are only slightly shifted with respect to each other. However, the intramolecular interactions of this Y within the respective PUB domain differ (Figure 7 and Figure S3). In contrast to PNGase-Y51, UBXD1-Y194 is involved in a potential hydrogen bonding network with the short loop between helices α_2_ and α_3_. In UBXD1, the OH group of Y194 lies in H bonding distance (2.9 Å) to the carboxyl group of E191, which likely also forms an H bond to H188 (2.8 Å). In place of UBXD1-E191, PNGase features D48 with a shorter side chain, which is also more turned away from the conserved tyrosine (Y51 in PNGase). PNGase-Y51 is placed slightly lower than UBXD1-Y194, increasing the distance to PNGase-D48 further. In addition, H188 in UBXD1 is replaced with N45 in PNGase, which is not in H bonding distance to PNGase-D48. This H bonding network, which is missing in PNGase, explains the local structural destabilization of the UBXD1-Y194 mutations observed in the CD spectrum and their moderate loss in PIM binding affinity. In contrast, the corresponding PNGase K50A, Y51A double mutant did not show impaired binding in a pulldown assay [29]; however, this assay is only qualitative in nature and does not reflect small changes in binding affinity.

In PNGase-PUB, the NH_2_ group of the conserved N41 hydrogen bonds the OH group of p97-Y805 (2.8 Å). In our UBXD1-PUB model, the corresponding N184 shows an alternate rotamer with the side chain carbonyl facing the OH group of p97-Y805, which possibly is an artifact because there were not sufficient NOE distance restraints to define the rotamer in the apo structure. This results in a larger potential H bond distance (3.2 Å) compared to the PNGase complex and brings the p97-Y805-OH group closer, and possibly into H bonding distance (3.2 Å), to the backbone carbonyl of UBXD1-Y181 (Figure 7). The p97-L804 side chain is bound in an almost identical conformation in both complexes, in a hydrophobic pocket formed by the conserved UBXD1-Y181/PNGase-Y38 and the hydrophobic side chain portions of T177 and K180 in UBXD1 and L34 and T37 in PNGase.

A conserved asparagine (N201 in UBXD1 and N58 in PNGase) is in hydrogen bonding distance to the C-terminal carboxylate of p97-G806 in both protein complexes (2.9 Å in both complexes). Additionally, the C-terminal carboxylate of p97 is in close contact to the NHε atom of PNGase-R55, likely forming a salt bridge. Upon mutation of this residue, p97-PIM binding is completely abolished in PNGase-PUB [25]. In contrast, the corresponding K198 in the UBXD1 apo structure forms an intramolecular salt bridge to D232 situated in the L1 loop extension, not present in PNGase, which is still present in our complex model (Figure 6 and Figure 7). Even though K198 is positioned such that it could possibly reach p97-G806 after a conformational change of its side chain and release of the contact to D232, it does not seem likely judging from the only moderate 3.5-fold effect on binding when this position is mutated in UBXD1-PUB.

Out of the stretch of negatively charged residues in p97-PIM, only two (D802 and D803) are visible in the PNGase/p97 complex crystal structure, with the remaining ones being disordered in the crystal; therefore, only these two aspartates are included in our model. In PNGase-PUB, R64 forms a potential salt bridge to p97-D802 (2.6 Å, Figure 7), while the corresponding R207 in our UBXD1-PUB complex model remains in a rotamer where it does not contact p97-D802. However, this could be a bias from the apo structure, and it is possible that R207 changes its conformation to contact p97-D802 on a time scale beyond our energy minimization. The six-fold reduction in binding affinity of the R207E mutant shows that this residue has some importance, but from our model it is not certain whether this is based on a direct contact to p97-D802 or on the overall positive electrostatic potential to which R207 contributes. The second p97 aspartate, D803, stabilizes the bound conformation of the p97 peptide in both complexes through a putative intramolecular H bond to the backbone amide HN of p97-G806 (Figure 7).

HOIP-PUB features a slightly different YxxN(x)_6_PxY motif where the P contacts the PIM tyrosine, which results in the p97-PIM sitting slightly higher in the pocket compared to UBXD1 and PNGase (Figure S4). The nearby Y faces the PIM pocket in the apo structure, but is turned away in the structure of HOIP bound to p97-PIM. UBXD1, PNGase, and HOIP also share the K/RxxN motif (K198 and N201 in UBXD1), which binds the free C-terminal carboxylate of p97-G806. HOIP, however, can also bind the OTULIN- and SPATA2-PIMs, which are situated within the protein sequence. The overall positive surrounding of the PIM pocket serves to bind the negatively charged residues preceding the PIM tyrosine. Overall, the UBXD1 PIM pocket exhibits the conserved features also observed in PNGase- and HOIP-PUB. In contrast, SPATA2-PUB has not been found to bind a PIM sequence. The PIM pocket lacks the first conserved tyrosine, which is replaced by valine, and also shows a slightly different arrangement of the conserved residues. Instead, SPATA2-PUB binds the USP domain of CYLD, which dimerizes via its B-box and does not contain a PIM sequence [28].

UBXD1, PNGase, and HOIP PUB domains bind the p97-PIM peptide with K_D_s in the low µM range and a 1:1 stoichiometry (Table 2, Figure 4, Figure 5 and Figure S2, and [25,27,34]). The K_D_ of HOIP for the OTULIN- and SPATA2-PIMs, however, is 50 times lower and lies in the nM range, which maybe reflects that these complexes either operate at lower concentrations in the cell or a have a lesser need to exchange their binding partners. p97, on the other hand, might need to swap cofactors more readily either within one pathway or to switch between different pathways.

The affinities of the isolated PUB domains of UBXD1 (Table 2, Figure 3D) and PNGase for full-length p97 lie in the same range as for the isolated PIM peptides. While for the isolated PNGase-PUB with p97, a 1:1 stoichiometry has been found, and only two full-length PNGase can bind to one p97 hexamer [25], likely because of steric hindrance. In the case of UBXD1, at least in the endo-lysosomal damage response (ELDR), which regulates autophagy of lysosomes, it binds p97 concomitantly with another cofactor PLAA that also binds the p97 C-terminus [16], which also implies that not all PIM sites within the p97 hexamer are bound by UBXD1.

Phosphorylation of p97-Y805 completely abolishes binding of the p97-PIM to UBXD1-PUB (Figure S1B), which has also been reported for the interactions of PNGase and HOIP with their respective PIM motifs [25,34]. This observation further supports the binding mode derived from our model (Section 3.3) where the Y residue is inserted into a hydrophobic pocket and likely stabilized by aromatic stacking and H bonding interactions. The Y hydroxyl group is completely buried in this model. Phosphorylation at this position adds additional bulk that cannot be accommodated by the size of the pocket, and it also introduces a negative charge that cannot be stabilized in a hydrophobic environment. Most likely, phosphorylation of the conserved tyrosine within all PIM motifs serves a regulatory function so that the PUB–PIM interactions can be switched on or off (e.g., to regulate p97 activity during ERAD) [26].

### 4.3. PUB Domains as Interaction Hubs in the Ubiquitin–Proteasome System

PUB domains (Figure 6) bind to PIM-containing proteins like p97 and other members of the UPS via their conserved PIM-binding pocket, resulting in a modular assembly of these protein complexes. Binding is abolished upon PIM phosphorylation, which allows regulation of the protein degradation machinery in response to cellular damage [26]. Some PUB-containing proteins like PNGase have enzyme activity, opening up the possibility that they could process substrates emerging from the D2 pore of p97 after unfolding. However, UBXD1 lacks such activity and may execute a regulatory role.

Interestingly, the PUB domains of SPATA2 and PNGase show different or additional protein interaction modes: SPATA2 binds to the USP domain of the deubiquitinase CYLD involving residues of the PIM pocket, even though CYLD does not contain a PIM motif [28]. This interaction is dependent on the dimerization of CYLD through its B-box zinc finger domain [28].

The PIM pocket of SPATA2-PUB differs from the canonical pocket and does not bind any of the known PIM sequences, but it is involved in binding the PIM-less CYLD, indicating that this interaction follows an alternative mode that is not compatible with PIM binding at the same time. In contrast, the PNGase PUB domain can interact with the UBL domain of proteasome shuttle factor HR23 involving the turn between sheets β_2_ and β_3_ and the shorter helices α_3_ and α_4_ (corresponding to loop L1 and helices α_3_ and α_4_ in UBXD1-PUB) [49]. This interaction mode leaves the PIM binding pocket available, thereupon Kamiya et al. [58] propose that p97-bound PNGase can bind, and thus activate, HR23 at the same time. These two examples suggest that PUB domains function not just as PIM motif-binding adapters but constitute protein interaction hubs within their respective Ub-processing complex that are important for complex assembly and its regulation. Thus, UBXD1, through its PUB domain, may have a key role in organizing interactions with additional proteins that act at the C-terminus of p97 downstream of substrate unfolding and, at the same time, functionally link this to regulation of p97-N-domain movements and ATPase activity though the second binding site in UBXD1. Alternatively, the PUB domain could possibly mediate intramolecular contacts in the context of full-length UBXD1, which might help to orchestrate the binding mode (just the N-terminus, just PUB, or both) of UBXD1 to p97 in a temporal fashion during the p97 ATPase cycle or depending on the presence or absence of other cofactors. Our structure of the UBXD1-PUB domain may now help with further work to unravel the interaction network and understand its relevance for disease pathogenesis.

## Figures and Tables

**Table 1:** Input for the structure calculation (constraints) and characterization of the ensembles of 20 energy-minimized CYANA conformers used to represent the NMR structure of UBXD1-PUB (150–264).

Parameter		Value
Total structures computed		100
Number of structures analyzed		20
*Constraints*		
NOE-based distance constraints		
Total		1221
intra-residue [i = j]		462
sequential [| I − j | = 1]		352
medium range [1 < | i − j | < 5]		211
long range [| i − j | ≥5]		196
NOE constraints per restrained residue		10.3
Dihedral angle constraints		567
Total # of restricting constraints		1788
Total # of restricting constraints per residue		15.0
Restricting long-range constraints per residue		1.6
*Residual constraint violations^a)^*		
Distance violations / structure		
0.1–0.2 Å		2.3
0.2–0.5 Å		0.2
>0.5 Å		0
RMS of distance violation / constraint		0.02 Å
Maximum distance violation		0.21 Å
Dihedral angle violations / structure		
1°–10°		2.6
>10°		1.05
RMS of dihedral angle violation / constraint		1.34°
Maximum dihedral angle violation		36.2°
*RMSD to the mean structure*		
	all	ordered
All backbone atoms	1.6 Å	0.9 Å
All heavy atoms	1.8 Å	1.3 Å
*Ramachandran plot statistics ^b)^*		
Most favored regions		85.9%
Additionally allowed regions		13.3%
Generously allowed regions		0.2%
Disallowed regions		0.5%

^a)^ after energy minimization with experimental constraints in Yasara. ^b)^ from Procheck.

**Table 2:** Dissociation constants (K_D_) of UBXD1-PUB mutants with p97-C10 peptide or full-length p97 (p97-fl) determined by fluorescence anisotropy.

Mutant	K_D_ (µM)
wild type (wt)	13.8 ± 0.3
Y181A	111 ± 2
Y181F	28.4 ± 0.6
N184A	28.7 ± 0.5
N184D	80 ± 2
K193E	17.7 ± 0.4
Y194A	53 ± 2
Y194F	141 ± 2
K198E	49.3 ± 0.8
Q200A	8.3 ± 0.3
N201A	64.1 ± 0.8
N201D	280 ± 3
V203A	25.3 ± 0.5
E206R	39.4 ± 0.9
R207E	88 ± 1
wt + p97-fl	8.4 ± 0.2

## Supplementary Materials

The following are available online at https://www.mdpi.com/2218-273X/9/12/876/s1, Figure S1: ^15^N-HSQC titrations of ^15^N-UBXD1-PUB with the p97-C10 or the phosphorylated p97-C13 peptide. Figure S2: Fluorescence anisotropy titrations for all UBXD1-PUB mutants. Figure S3: Superposition of UBXD1-PUB and PNGase-PUB apo structures showing the H-bonding network of UBXD1-Y194. Figure S4: Superposition of the UBXD1-PUB/p97-PIM model and the crystal structures of PNGase and HOIP in complex with p97-PIM showing the position of the PIM peptide. Table S1: Oligonucleotides used as primers for PCR-amplification of the UBXD1-PUB (150-264) construct and for site-directed mutagenesis following the Quikchange protocol.
