# Supplementary material for: Structure of the PUB Domain from Ubiquitin Regulatory X Domain Protein 1 (UBXD1) and Its Interaction with the p97 AAA+ ATPase
Source: Biomolecules. 2019 Dec 14;9(12):876. doi: 10.3390/biom9120876 (PMC6995525; doi:10.3390/biom9120876)
Supplement: Supplementary file 1 [file biomolecules-09-00876-s001.pdf]

biomolecules

Structure of the PUB domain from Ubiquitin Regulatory X domain protein 1 (UBXD1) and its interaction with the p97 AAA+ ATPase

Mike Blueggel[1], Johannes van den Boom[2], Hemmo Meyer[2], Peter Bayer[1] and Christine Beuck[1,*]

1 University Duisburg-Essen, Structural and Medicinal Biochemistry, Centre for Medical Biotechnology (ZMB), 45117 Essen, Germany.
2 University Duisburg-Essen, Molecular Biology, 45117 Essen, Germany.
* Correspondence: christine.beuck@uni-due.de; Tel.: +49-201-183 2929.

Supplemental Material

- **Figure S1**: ^{15}N-HSQC-NMR titrations of UBXD1-PUB with p97 PIM peptides
- **Figure S2**: Fluorescence anisotropy titrations for all UBXD1-PUB mutants.
- **Figure S3**: Superposition of UBXD1-PUB and PNGase-PUB apo structures showing the H-bonding network around UBXD1-Y194 that is not present in PNGase.
- **Figure S4**: Superposition of the UBXD1-PUB/p97-PIM model and the crystal structures of PNGase and HOIP in complex with p97-PIM showing the position of the PIM peptide.
- **Table S1.** Oligonucleotides used as primers for PCR-amplification of the UBXD1-PUB (150-264) construct and for site-directed mutagenesis following the Quikchange protocol.

Figure S1

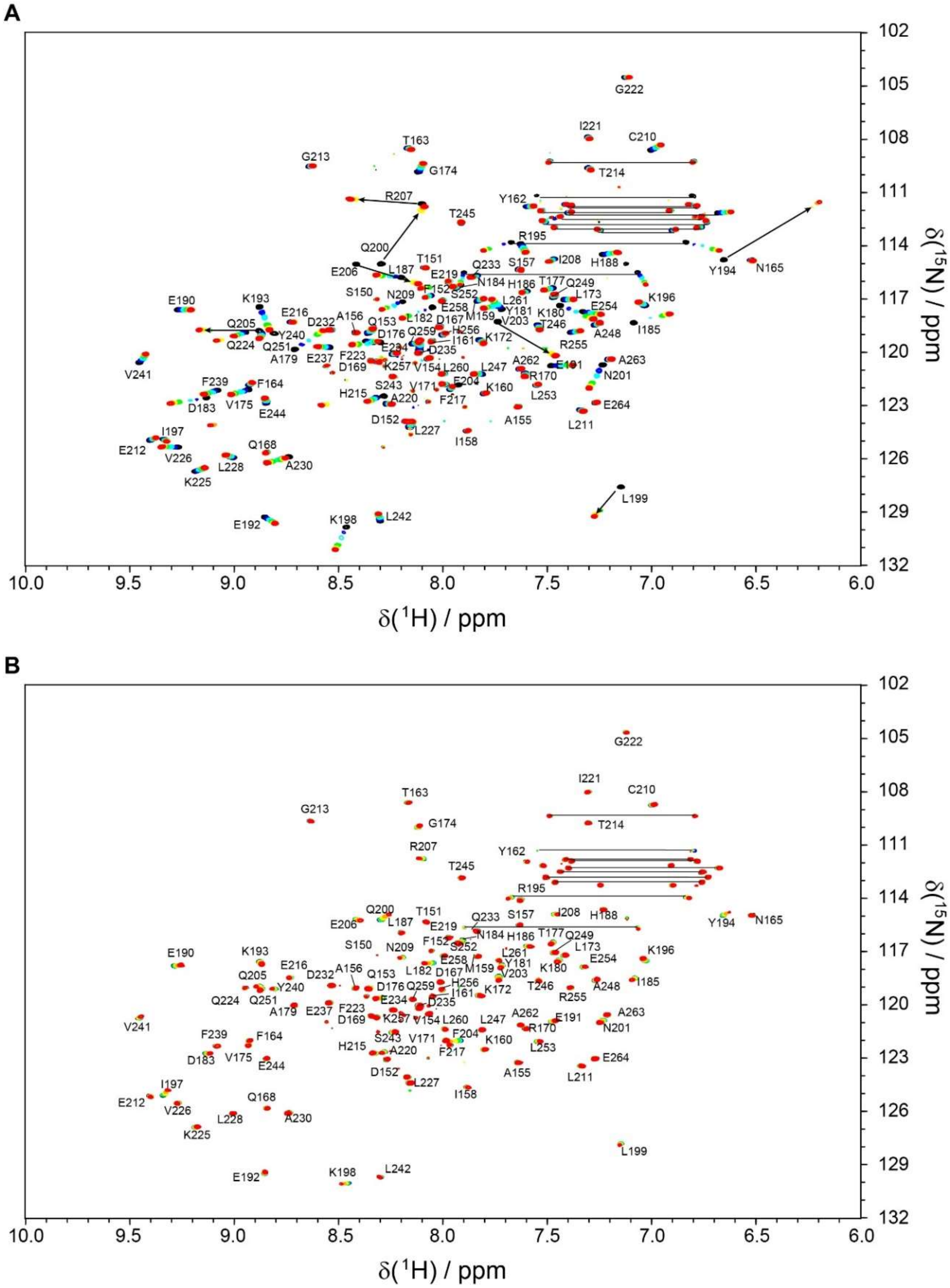

Figure S1. ^{15}N-HSQC-NMR titrations of UBXD1-PUB with p97 PIM peptides. (**A**) ^{15}N-HSQC titration of ^{15}N-labelled UBXD1-PUB with p97-C10 PIM peptide. Peptide concentrations range from 0 μM (black) to 1.1 mM (red). Peaks shift during the titration, indicating intermediate-to-fast-exchange. The trajectories of signals which experience line broadening due to intermediate exchange are indicated with an arrow; (B) ^{15}N-HSQC titration of ^{15}N-labelled UBXD1-PUB with phosphorylated p97-C13 PIM peptide shows no binding. Peptide concentrations range from 0 μM (black) to 1.1 mM (red).

Figure S2

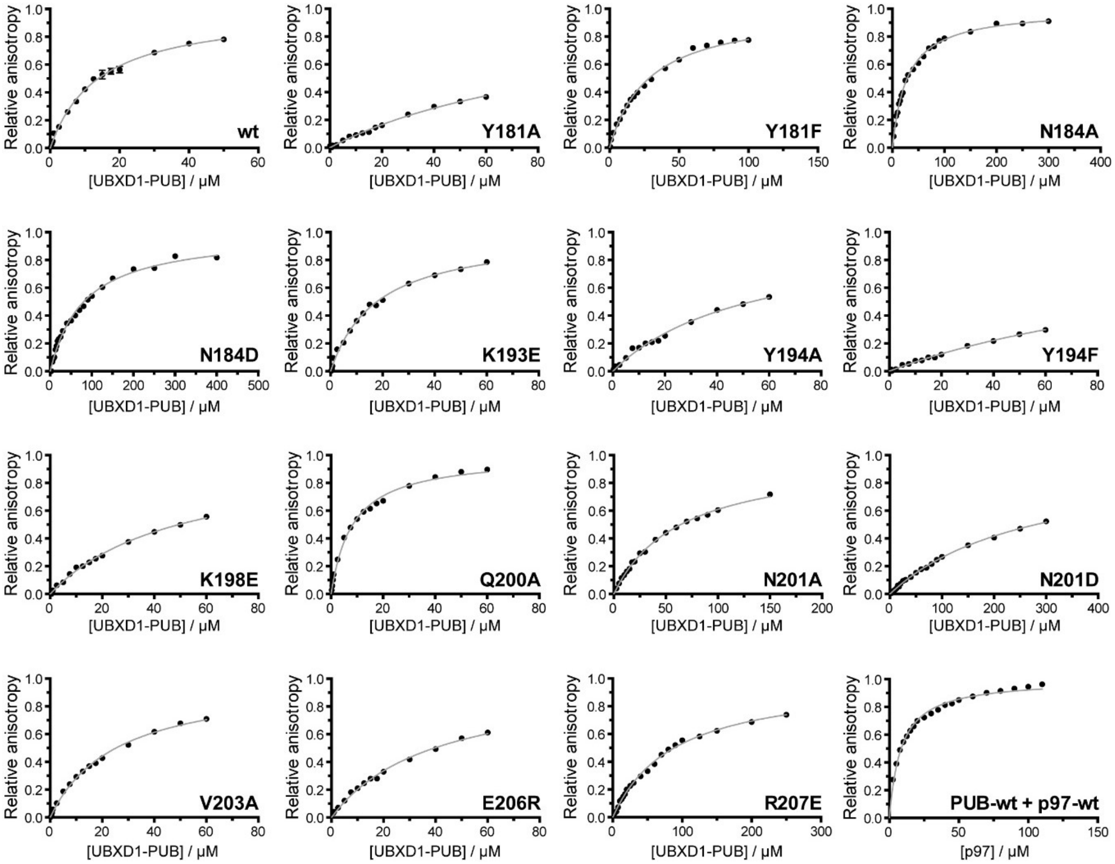

Figure S2. Fluorescence anisotropy binding curves of FAM-labeled p97-C10 peptide with UBXD1-PUB mutants and of UBXD1-PUB with full length p97. Error bars represent the standard deviation of three experiments.

Figure S3

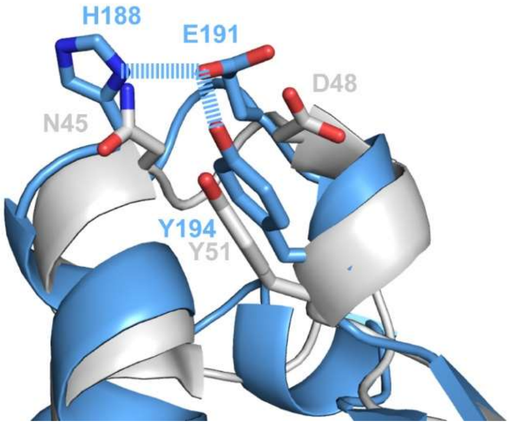

Figure S3. Superposition of the UBXD1-PUB (blue) and PNGase-PUB apo (gray, pdb # 2CCQ, [29]) structures showing the intramolecular H-bonding network (blue dashed lines) around UBXD1-Y194 that is not present in PNGase.

Figure S4

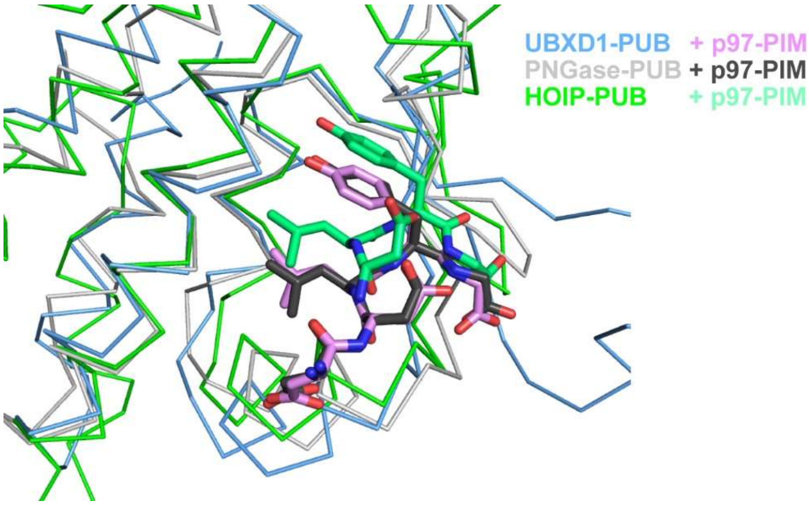

Figure S4. Superposition of the UBXD1-PUB/p97 complex model (UBXD1-PUB backbone in blue, p97-PIM in pink) with the crystal structures of PNGase/p97-PIM (PNGase-PUB backbone in gray, p97-PIM in black) and HOIP-PUB/p97-PIM (HOIP-PUB backbone in bright green, p97-PIM in mint green).

Table S1

Table S1. Oligonucleotides used as primers for PCR-amplification of the UBXD1-PUB (150-264) construct and for site-directed mutagenesis following the Quikchange protocol.

Construct	Oligonucleotides
UBXD1-PUB (150-264) wt	CAGTCATATGTCCACCGACCCAG<br>TCACAAGCTTTTATCACTCCGCAGCCAGCAG
**Mutant**	
Y181A	CCATTGCCAAGGCGCTGGACAACATCC<br>GGATGTTGTCCAGCGCCTTGGCAATGG
Y181F	CCATTGCCAAGTTCCTGGACAACATCC<br>GGATGTTGTCCAGGAACTTGGCAATGG
N184A	CAAGTACCTGGACGCCATCCACCTGCAC<br>GTGCAGGTGGATGGCGTCCAGGTACTTG
N184D	CAAGTACCTGGACGACATCCACCTGCAC<br>GTGCAGGTGGATGTCGTCCAGGTACTTG
K193E	CCGAGGAGGAGGAGTACCGGAAGATC<br>GATCTTCCGGTACTCCTCCTCCTCGG
Y194A	CGAGGAGGAGAAGGCACGGAAGATCAAGC<br>CGAGGAGGAGAAGGCACGGAAGATCAAGC
Y194F	GAGGAGGAGAAGTTTCGGAAGATCAAGC<br>GCTTGATCTTCCGAAACTTCTCCTCCTC
K198E	GTACCGGAAGATCGAGCTGCAGAAC<br>GTTCTGCAGCTCGATCTTCCGGTAC
Q200A	GGAAGATCAAGCTGGCGAACAAGGTGTTTC<br>GAAACACCTTGTTCGCCAGCTTGATCTTCC
N201A	GATCAAGCTGCAGGCCAAGGTGTTTCAGG<br>CCTGAAACACCTTGGCCTGCAGCTTGATC
N201D	GATCAAGCTGCAGGATAAGGTGTTTCAGG<br>CCTGAAACACCTTATCCTGCAGCTTGATC
V203A	CTGCAGAACAAGGCGTTTCAGGAGCG<br>CGCTCCTGAAACGCCTTGTTCTGCAG
E206R	CAAGGTGTTTCAGCGCCGCATTAACTGCC<br>GGCAGTTAATGCGGCGCTGAAACACCTTG
R207E	GGTGTTTCAGGAGGAGATTAACTGCCTGG<br>CCAGGCAGTTAATCTCCTCCTGAAACACC
